# Telehealth Adoption During COVID-19: Lessons Learned from Obstetric Providers in the Rocky Mountain West

**DOI:** 10.1089/tmr.2023.0001

**Published:** 2023-02-24

**Authors:** Carly Holman, Annie Glover, Kimber McKay, Courtney Gerard

**Affiliations:** ^1^ Rural Institute for Inclusive Communities, University of Montana, Missoula, Montana, USA.; ^2^ School of Public and Community Health Sciences, University of Montana, Missoula, Montana, USA.

**Keywords:** COVID-19, obstetric care, rocky mountain west, rural, telehealth

## Abstract

**Introduction:**

Obstetric providers have used telemedicine to manage gestational diabetes, mental health, and prenatal care. However, the uptake of telemedicine in this field has not been universal. The COVID-19 pandemic catalyzed the adoption of telehealth in obstetric care, which will have lasting effects, especially for rural communities. We sought to understand the experience of adapting to telehealth among obstetric providers in the Rocky Mountain West to identify implications for policy and practice.

**Methods:**

This study included 20 semi-structured interviews with obstetric providers in Montana, Idaho, and Wyoming. The interviews followed a moderator's guide based on the Aday & Andersen Framework for the Study of Access to Medical Care, exploring domains of health policy, the health system, the utilization of health services, and the population at risk. All the interviews were recorded, transcribed, and analyzed using thematic analysis.

**Results:**

Findings indicate that participants view telehealth as a useful tool during prenatal and postpartum care; many participants intend to continue telehealth practices after the pandemic. Participants shared that their patients reported benefits to telehealth beyond COVID-19 safety, including limiting travel time, reducing time off work, and alleviating childcare needs. Participants expressed concern that expanding telehealth will not equally benefit all patients and could widen existing health inequities.

**Discussion:**

Success moving forward will require a telehealth infrastructure, adaptive telehealth models, and provider and patient training. As obstetric telehealth expands, efforts must prioritize equitable access for rural and low-income communities, so all patients can benefit from the technological advancements to support health.

## Introduction

Before the 2019 novel coronavirus (COVID-19) pandemic, the use of telehealth in obstetric care varied among providers with limited implementation.^1^ The onset of COVID-19 led to the rapid adoption of telehealth across health systems, including obstetric care. The resulting changes in reimbursement mechanisms, Health Insurance Portability and Accountability Act regulations, and approved communication modes (video, phone, text messaging) transformed the delivery of perinatal care.^2^

Obstetric providers' experiences during this time can advance the field by informing key improvements to ensure the long-term utility of telehealth services.^1,3^ This study sought to understand obstetric providers' experiences transitioning to telehealth to identify implications for policy and practice to ensure continued uptake, use, and sustainability for the COVID-19 pandemic and beyond.

On March 11, 2020, the World Health Organization declared COVID-19 a global pandemic.^4^ As states underwent shelter-in-place orders, health systems suspended and postponed non-essential and elective medical care and shifted to telehealth to reduce exposure for patients and providers.^3^ A core element of severe public health emergencies involves managing care for high-risk populations.^5^ Respiratory illnesses disproportionally affect pregnant people and their fetuses, putting them at greater risk of COVID-19.^5^

As the pandemic progressed, more information became available on the association between pregnant people with COVID-19 and higher rates of adverse outcomes, including maternal mortality, preeclampsia, and preterm birth.^6^ The considerable risk of severe maternal morbidity and mortality, and neonatal complications further underscored the need to enhance virtual care models to protect patients.^6^

The use of telehealth in obstetric care has demonstrated effectiveness as a general maternal health delivery tool^7,8^ as well as in specialty applications, such as gestational diabetes,^9^ management of hypertensive disease,^10^ mental health,^11,12^ breastfeeding,^13,14^ and prenatal care.^15^ The Obstetric (OB) Nest program has successfully implemented a reduced-frequency prenatal care model utilizing telehealth, resulting in high levels of patient satisfaction.^15^

Since the onset of the pandemic, multiple studies^16–19^ have demonstrated that for many obstetric patients, particularly working mothers and multiparous mothers, the convenience of telehealth made it a desirable alternative to in-person care. Previous studies indicate a lack of hesitation with patient adoption and a comparable degree of patient satisfaction.^15,20^

Although telehealth interventions have generally shown positive impacts in obstetric care, it does not come without challenges. Telehealth requires substantial investment in equipment, technology, and staffing, making it difficult for small and under-resourced clinics to meet financial demands.^21^ Some patient populations face additional barriers, including access to reliable broadband, smartphones, and equipment to support remote monitoring.^1,22^ Without thoughtful planning, telehealth can widen disparity gaps and exacerbate existing inequities in the health care system.^22,23^

The rapid shift to telehealth in response to COVID-19 did not permit time for careful planning on its implementation.^24^ Through qualitative interviews with obstetric providers from the rural Rocky Mountain West (Montana, Idaho, and Wyoming), this study aimed to (1) describe the experiences of obstetric providers moving to telehealth models during a pandemic and (2) generate clinical delivery recommendations for systems to institutionalize telehealth in obstetric care in rural communities during the pandemic and beyond.

Given the central issue of access to this delivery innovation, we used Aday & Andersen's Framework for the Study of Access to Medical Care as theoretical guidance in this qualitative analysis.^25^ The framework outlines conditions that either facilitate or hinder use. These conditions are organized within five components: health policy, characteristics of the health delivery system, utilization of health services, characteristics of the population at risk, and consumer satisfaction.^25^

## Methods

### Participants

This study included semi-structured qualitative interviews with obstetric providers, obstetrician/gynecologists, maternal-fetal medicine specialists, and certified nurse-midwives in Montana, Idaho, and Wyoming. We selected these states due to their geographic similarities, with most counties in each state classified as rural. We used a purposive, non-probabilistic sampling methodology.^26^

Recruitment occurred through the American College of Obstetricians and Gynecologists District VIII listserv, mailings, and phone calls to hospitals and health clinics. Interested providers contacted a research team member to schedule an interview. The Institutional Review Board approved the study under Protocol # 104-20.

### Data collection

Data collection occurred from July 2020 to September 2020. C.H. conducted all interviews over Zoom. At the beginning of each interview, participants provided informed consent. The interviews followed a semi-structured moderator's guide. The Aday & Andersen Framework for the Study of Access to Medical Care^25^ informed the development of questions exploring domains that related to enabling health policy, characteristics of the health delivery system (resources, equipment, training), utilization of health services (type of service, site, and purpose), and population characteristics (that may impact telehealth utilization).

We did not address the consumer satisfaction component of the framework, as we did not conduct interviews with obstetric patients. All interviews were recorded (duration range 17–39 min) and transcribed. Data collection continued until reaching saturation at 20 interviews, with no new themes emerging.

### Data analysis

We conducted a hybrid inductive/deductive process for coding and theme development. One member of the research team C.H. reviewed the transcripts and organized an initial set of themes into a codebook. The coding scheme followed the interview question content areas. C.H. continually re-read transcripts and updated the codebook as new themes emerged. Next, a team of three coders K.M., A.G., and C.G., with doctoral-level training in qualitative analysis, reviewed the transcripts and made updates to the codebook through an inductive process.

Once the group agreed on the final codebook, K.M., A.G., and C.G., each independently coded all the transcripts. The coders reviewed the coded transcripts and repeatedly met to discuss coding discrepancies and revise codes. The research team then came to a consensus on the major themes and their significance to the research questions. The data were analyzed using MAXQDA 2020.^27^

The interview guide gathered self-reported information on participant characteristics, including their profession, geographic location of their practice, hospital affiliation, years in practice, and experience with telehealth in their practice. We categorized these responses to provide an overview of participant characteristics (Tables 1 and 2).

**Table 1.** Participant Characteristics

Participant characteristics (***N*** = 20)	***n*** (%)
Profession	
Obstetrician	15 (75)
Maternal-fetal medicine specialist	3 (15)
Certified nurse midwife	2 (10)
Geographic location of practice	
Noncore	5 (25)
Micropolitan	7 (35)
Small metro	4 (20)
Medium metro	4 (20)
Affiliated hospital	
Hospital (non-CAH)	15 (75)
CAH^a^	5 (25)
State	
Montana	9 (45)
Idaho	7 (35)
Wyoming	4 (20)
Years in practice	
1–10 Years	8 (40)
11–24 Years	8 (40)
25+ Years	4 (20)

^a^ Rural hospitals designated as CAHs by the Center for Medicare & Medicaid Services.

CAH, critical access hospital.

**Table 2.** Telehealth Implementation by Participants Before, During, and After COVID-19

Telehealth implementation (***N*** = 20)	***n*** (%)
Before COVID-19	
Used telehealth	4 (20)
Did not use telehealth	16 (80)
During COVID-19	
Used telehealth	18 (90)
Did not use telehealth	2 (10)
Plans for after COVID-19	
Continue using telehealth	17 (85)
Will not use telehealth	3 (15)

We categorized the geographic location of their practice using the National Center for Health Statistics (NCHS) Urban-Rural Classification Scheme for Counties (noncore, micropolitan, small metro, and medium metro).^28^ Hospital affiliation included hospital or critical access hospital, and we grouped years in practice as (1–10, 11–24, and 25+ years). We organized the responses about the interviewee's use of telehealth into three time periods, including (1) use of telehealth before COVID-19, (2) use of telehealth during COVID-19, and (3) plans to use telehealth after COVID-19 (Table 2).

## Results

### Participant characteristics

Twenty providers participated in the study, with nine practicing in Montana, seven in Idaho, and four in Wyoming. Obstetricians comprised most participants (75%), followed by maternal-fetal medicine specialists (15%) and certified nurse-midwives (10%). Participants had various experiences, from recently out of residency to approaching retirement (Table 1). Most participants (80%) had not utilized telehealth before the onset of COVID-19. This shifted dramatically during the pandemic, with almost all (90%) using telehealth to deliver care. As participants forecasted to post COVID-19, most (85%) thought they would continue to use telehealth in their practice (Table 2).

The following sections present the themes revealed through the qualitative data analysis. We organized the themes within four components of the Aday & Andersen Framework described earlier: health policy, health delivery system, utilization, and the population at risk.^25^ The health policy component included one theme, *equitable access to equipment and technology*. Four themes aligned with the characteristics of the health delivery system: *administrative support*, *equipment and technology*, *changes in workflow*, *and communication with patients*. Utilization of Health Services included two themes *type of care* and *patient engagement with telehealth*, and lastly, characteristics of the population at risk had one theme *access to care*.

### Health policy

The theme *equitable access to equipment and technology* included three subthemes, access, connectivity, and in-home monitoring (Table 3). Eighteen participants shared that telehealth worked well for patients with access to the appropriate devices and technology. However, this did not account for their entire patient population.

**Table 3.** Health Policy Themes

Theme	Sub-themes	Examples of participant quotes
Equitable access to equipment and technology	Access	“I think that the only people that can do telehealth, are the people who have financial means. So, people who don't have internet connection, real country folk, and people who just don't have the money to have a camera on their computer or have a computer or have a smartphone. They're sort of out of luck.” [P16]
		“A lot of our patients don't have phones. They don't have computers, they don't have the internet, we don't have WiFi hotspots.” [P18]
	Connectivity	“Connection is a huge issue, there's a lot of our patients, especially in rural areas, that don't have access to high speed internet. Then even cell phone data connections can be hit or miss around here, so there's some visits where we would start with a video, and then the video didn't work, and it would cut out, and all of those things. We'd have to default to telephone. Yeah, I think that was an issue in the rural areas.” [P5]
		“So connectivity, we're not quite to the point in a rural area where you have high speed internet access 24/7. In the hospital it usually is, but especially for patients somewhere else, they're connectivity can be an issue.” [P17]
	In-home monitoring	“I mean, it's hard because the statistics I need don't really come from an Apple Watch. I need a blood pressure and a weight, and then a fetal heart rate… Apparently, there's some device that can measure the fetal heart rate pretty easily, and some of my patients have bought that and used it.” [P15]
		“The rate limiting thing for our patient population, just giving the low resource population that we serve is that sometimes patients can't afford to go and buy like a blood pressure cuff.” [P1]

Ten participants described barriers encountered by patients without computers or phones to support telehealth. Fourteen participants shared that in-home monitoring worked well but only served those who had the means to acquire the equipment. Twelve participants serving rural communities noted that limited broadband and poor internet connection posed barriers for patients to use telehealth services.

### Characteristics of the health delivery system

Four themes surfaced that align with the characteristics of the health delivery system, *administrative support*, *equipment and technology*, *changes in workflow*, and *communication with patients* (Table 4). The first theme highlighted the impact of *administrative support and guidance* on their transition to telehealth. Fourteen participants discussed how the support (or lack thereof) from administration and IT influenced the rollout of telehealth. Those who received direction and support had a much easier time adapting to virtual care.

**Table 4.** Characteristics of the Health Delivery System Themes

Theme	Sub-themes	Examples of participant quotes
Administrative support	Adequate support facilitated implementation	“Our IT folks did amazing, I think we went from turning it on, to beta testing it, to using it on patients in about 48 hours. Yeah. And there were a few kinks here and there to work out just in terms of how it worked and all that. But that was it. That was a huge shift. Just to have the capability of utilizing that.” [P10]
		“We all are intimately supported by the hospital, both in the clinic setting, as well as the hospital setting. It's all one unit so it actually was pretty efficient and instituted in a very timely fashion and the support was excellent.” [P9]
	Inadequate support created barriers to implementation	“When the idea of telehealth started in March, there really wasn't any support from administration or IT. I feel like this was totally foreign to anybody at our hospital, so they weren't really sure how to go about this.” [P5]
		“I think platform, having a platform that was universal and recommended or mandated by the facility, we were all just left to our own devices.” [P7]
Equipment and technology	Access	“So I have had to buy more cameras and, since that, I have put in another monitor with cameras and speakers in each of our locations so that we can do that more readily. So yeah, we've spent some money… Equipment mostly, we had Zoom.” [P12]
		“Yeah. I mean, they literally dropped off an iPad in addition to my computer, with instructions, and I just read the instructions, and we just started doing it with some trial and error, and some of it was really easy.” [P15]
	Telehealth platform	“Let's see, initially we tried to use Lifesize. That was the program that the hospital was using for all of our meetings and it was somewhat cumbersome. Then we went to… Oh, we used Skype. We did that for a while. And then we ended up using the one called Doxy.me through Doximity. That seemed to be the best one, because patients didn't have to download anything. They just went to a web address and were able to sign on that way.” [P11]
		“Our EHR, they built it in real quick. Jipsu, I think is the one that they use. But it was just built into the schedule in our EHR. And so you just click the button and it popped into that.” [P17]
Changes in workflow		“Just adapting to not being able to see the patient in my usual environment, I guess. And I think that was … I was used to my routine of doing things and I had a way of doing things where I didn't miss stuff. And sometimes, I would forget to ask them questions and have to call them back up later.” [P6]
		“A lot of it just is the logistics, or just the mechanics, the mechanics of how our Telehealth works. So, I'm used to an office flow where the patient comes, they check in, the nurse puts them in a room, I have time to be doing other things. And then when they're ready, I go in and see them. With Telehealth, I'm the one that initiates that interaction with the way our EHR works.” [P10]
Communication with patients		“I use a little bit more confirmatory questioning, reflecting back to patients what they've asked or what they're concerned about, just to make sure that they're getting the message, right? That we're connecting, so that I close that loop and make sure I'm not missing something in their questions… Speaking slowly, more slowly than I'm talking to you very clearly and also leaving a lot of space in the conversation, so that if they did have a question they feel comfortable chiming in.” [P10] “I think if you have video, that's nice, at least you can still see facial expressions and that kind of thing. Just over the phone can be a little bit hard because you don't get any of that. But with video, at least, you can still see that, which is helpful.” [P4]

The second theme highlighted the role of *equipment and technology* in telehealth. Eleven participants discussed the technological infrastructure required to offer a broad range of care, including acquiring the necessary equipment and a telehealth platform. The third theme focused on *changes in workflow*. Eight participants described the additional logistics of a telehealth appointment and noted differences in appointment progression in telehealth compared with in-person. Several participants struggled to adapt to the new appointment flow, resulting in missed questions.

The fourth theme encompassed *communication with patients.* Eleven participants spoke of adaptations to their communication style over telehealth, including asking more questions, slowing down, and positioning themselves close to the camera.

### Utilization of health services

Two themes surfaced that aligned with utilization of health services (Table 5). The theme *type of care* included four subthemes prenatal care, postpartum care, diabetes care, and patient risk. Participants gave examples of adapting elements of prenatal, postpartum, and diabetes care for telehealth. Eighteen participants described shifting talk-based appointments to virtual care across the prenatal and postpartum period. They also identified areas unsuitable for telehealth, including interventions requiring physical contact and some high-risk patients. Seven participants talked about assessing the patient's needs and using that information to determine if a telehealth appointment was suitable.

**Table 5.** Utilization of Health Services Themes

Theme	Sub-themes	Examples of participant quotes
Type of care	Prenatal care	“So, particularly for obstetrics, the visits that typically wouldn't involve any labs or ultrasounds or any other things like that, those are the ones that we preferred to do over telehealth.” [P17]
		“So visits like the 12 weeks, 16 weeks, we did by phone; 20 weeks, the patient comes in for other anatomy and we have an OB visit face-to-face with them at that time. And then 24 weeks was by phone; 28 weeks, they came in for their 1-hour glucose and all of that. And then between 28 and 36, we kind of did every other visit or phone visits until about 36-week mark. And then pretty much been seeing patients from 36 weeks on every week.” [P20]
	Postpartum care	“There were some postpartum visits as well. If they were a vaginal delivery without complications, we would do a lot of the two week visits that way.” [P11]
		“Then we would offer postpartum patients to be seen inpatient or telehealth.” [P13]
	Diabetes care	“I take care of a lot diabetes. And so it's easier to do diabetes care through it because I don't have to examine a patient. I just really need to look at their numbers and talk to them. So it's made access easier for our patients.” [P3]
		“I think it made some of my diabetic management where again, they didn't have to be seen in the clinic and I could manage their blood sugars by seeing their numbers in their monitor remotely, made that very smooth to make adjustments on their insulin regimens.” [P19]
	Patient risk	“We determined which patients were again, more routine and which patients were more of a high-risk and then determined which visits they could do in person and which visits we wanted them to do over the phone.” [P19]
		“For some of those where we could do a depression screening and they weren't having any physical concerns, then that could move to telemedicine. But it involved more trying to, for low risk patients particularly, space out some of the in-person visits, as long as they were feeling normal fetal movement, and there weren't other concerns.” [P4]
Patient engagement with telehealth	Convenience	“I had a couple who really liked it and who said, “Yeah, this is nice, it's so convenient.” So certainly there are some who definitely find that's the case.” [P4]
		“We've had some patients ask, “Hey, can I do that next visit on the phone? Because that was great.” They really do like it because really, obstetrical care, it gets a little bit onerous as you go along. And especially if you're traveling from far, having to come every every two weeks, and then every week at the end of pregnancy, and every month upfront. So I know some patients have enjoyed it.” [P8]
	Prefer in person	“Some patients were basically, “I'm not going to do that.” I'm just going to wait until I can come in.” [P4]
		“Some patients have really, really felt like they would rather come into the office despite the concerns around COVID.” [P8]
	Digital literacy	“I would say, a lot of our patients that could have taken advantage of telehealth, have not really been able to. And I think part of that is just… it takes so much training and practice to get people logged in reliably over the phone or over video, to sort of do some of these remote visits.” [P15]
		“Getting patients to understand how to do it. So it's just trying to… Some reason it seems like there's connection problems and trying to get people to answer their emails and trying to get people to understand how to do it is probably the biggest challenge.” [P3]

The second theme *patient engagement with telehealth* had three sub-themes, convenience, digital literacy, and preferred in-person. Eighteen participants shared that patients reported benefits to telehealth, such as limiting drive time to appointments, reduced time off work, and alleviating childcare needs.

Twelve participants reported that some patients would have used telehealth but struggled with digital literacy, negatively impacting their ability to engage with the technology. Although many participants spoke of patients' positive experiences, patient engagement with telehealth differed, eight participants discussed patients preferring in-person visits.

### Characteristics of the population at risk

One theme emerged that aligns with the characteristics of the population at risk and provides information on the factors that could impact individual determinants of utilization. It is important to state that the theme in this section represents participating providers' perspectives on their patient population. The theme *access to care* has three sub-themes, distance to care, transportation, and uninsured or underinsured (Table 6).

**Table 6.** Characteristics of the Population at Risk Themes

Theme	Sub-themes	Examples of participant quotes
Access to care	Distance to care	“We have patients 400 to 500 miles away that are economically challenged and have trouble getting down here to see us.” [P3]
		“And when they live two to three hours away, it's just not possible for them to come in twice a week.” [P16]
	Transportation	“So especially in the winter time, getting through the mountain passes can be challenging and sometimes just due to low resources, sometimes they don't have transportation to get to care or since because of weather.” [P1]
		“Yeah. I mean, a lot of my patients… are from the lower socioeconomic status. And so, they're relying on family members to drive them, an hour, to come to visits and then an hour back.” [P15]
	Uninsured or underinsured	“I just was discharging a mom this morning, who from a patient care perspective, her biggest challenge is just financial. So she didn't get hardly any prenatal care.” [P12]
		“There is a segment of uninsured or under insured people. So finding ways when they show up in your clinic with a gynecologic problem to take care of that.” [P4]

Fifteen participants discussed how the vast geographic landscape presents barriers for patients to access obstetric care, including travel time and winter driving conditions. Fourteen participants described transportation barriers encountered by patients, noting access to a reliable vehicle, money for gas, and availability of friends or family for rides. Five participants also shared that some patients face barriers to accessing care due to being uninsured or underinsured.

## Discussion

The COVID-19 pandemic changed the trajectory of obstetric care with the rapid adoption of telehealth. Despite minimal prior experience with telehealth, most providers who participated in this study (85%) anticipated continuing to integrate virtual care into their obstetric practice. Utilizing the Aday & Andersen Framework,^25^ we have organized recommendations from this study in the areas of health policy, characteristics of the delivery system, utilization of health services, and characteristics of the population at risk.

Health policy is the starting point in the Aday & Andersen framework for the study of access.^25^ A central component of health policy involves access to health services. Health policy can influence access to medical care through education, financing, health services, and insurance.^25^ The providers in this study saw many benefits to telehealth implementation; however, they expressed concern about whether it would equally benefit all patients.

Participants discussed challenges related to patient access to smartphones or laptops and broadband internet in some rural communities. If health systems continue to expand virtual care models, federal, state, and local governments must invest in infrastructure to reduce the digital divide.^22^ Doing so will allow all populations to benefit from expansions in telehealth equally.^2^ Participants emphasized the importance of in-home monitoring in telehealth obstetric care. During COVID-19, the ability to implement in-home monitoring depended on patient access to devices such as blood pressure cuffs and scales. Moving forward, payers need to cover the in-home monitoring equipment necessary for comprehensive prenatal care.^23^

Aday & Andersen conceptualize the health delivery system through two main components: resources (workforce, education/training, equipment) and organization (internal coordination of resources).^25^ The organization component also includes patient entry and navigation through the system.^25^

Several themes emerged from the interviews that highlighted the importance of telehealth infrastructure within the health system. Most participants reported learning the telehealth software as they went, with few receiving any formal training from their organization. Study participants had an easier time implementing telehealth when they received administrative support and guidance, including technical support from IT and direction on appropriate documentation.

Participants reported many challenges with adapting their clinical practice to deliver care remotely, most notably the changes to workflow. Telehealth appointments introduce an entirely new set of operations.^21,29^ Our findings have several implications for the development of telehealth delivery models. Health systems must create and adhere to a framework that standardizes patient enrollment and operations over telehealth.^21,29^ This will likely necessitate additional administrative staff time, and IT support to meet the needs of the providers and patients.^29^

In addition to logistical elements to support workflow, providers must receive training on adapting clinical practices to remote care.^24^ As telehealth expands, so does the need for education on patient-centered telemedicine and adapting communication styles to the virtual environment.^24,30^ Health systems must invest in training and prioritize telemedicine care delivery in continuing education and medical education.

Utilization of health services includes the type (medical service/provider), site (in-patient, out-patient, telemedicine), and purpose (preventative, illness-related, custodial care) of the medical care.^25^ Study participants reported that they could successfully shift many elements of prenatal and postpartum care to telehealth. Although they expressed some concern with high-risk patients, they felt telehealth had wide application in obstetric care.

OB Nest and other patient-centered models use in-person care for essential services, allowing patients to tailor additional care to meet their needs.^1,15^ High-risk patients can also benefit from telehealth models through specific modifications for increased surveillance and counseling.^31^ As obstetric telehealth expands, adopting a formalized in-person and virtual care model can support providers with streamlining care delivery for low-and-high-risk patients.^1^ The push of COVID-19 to change longstanding obstetric care models has resulted in a dramatic expansion of maternity care options, shifting agency to the pregnant person to determine their ideal care plan.^1^

The characteristics of the population at risk section of the framework capture the individual determinants of utilization. Aday & Andersen outline predisposing (age, race, sex, values), enabling (insurance, income, education level, community), and need (health status) components impacting access to medical care.^25^ Study participants discussed several enabling components that either facilitated or impeded their patients' access to obstetric care.

Many participants discussed that their patients live far from the health care facility, requiring patients pursuing in-person care to drive, in many instances, hundreds of miles. In winter, travel challenges are made especially acute by challenging road conditions. Coupled with low resources, some patients face barriers getting to appointments, especially as appointment frequency increases during pregnancy.

These factors contributed to patient interest in telehealth options. In addition, participants also discussed patient-level factors that impacted telehealth use, including access to a phone/computer with a video, in-home monitoring devices, sufficient bandwidth for connectivity, and digital literacy. Health care providers must consider unique patient characteristics such as preference, bandwidth/connection, and digital and health literacy when determining the appropriateness of telehealth for meeting patient needs.^3^

There are several limitations to this study. The findings are based on interviews with a small sample of providers who do not necessarily reflect the experience of other providers in the region. The interviews occurred within the first 6 months of the COVID-19 pandemic when providers had minimal experiences with telehealth; their perspectives could have changed later in the pandemic. Selection bias is also a limitation of this study, as providers responded to invites to participate in a study about telehealth in obstetric care.

Providers with more positive experiences with telehealth might have been more likely to participate. In addition, providers shared their views on patient experiences with telehealth. The provider's own implicit biases could influence these interpretations.

## Conclusions

The COVID-19 pandemic catalyzed the adoption of telehealth practices in obstetric care, which will have lasting effects, especially for rural communities. To our knowledge, this study is the first to use qualitative methods to understand the perspectives of providers implementing obstetric telehealth in sparsely populated states in the Rocky Mountain West.

This study highlights several opportunities and challenges in providing telehealth perinatal care. Long-term sustainability will require a telehealth infrastructure, adaptive telehealth models, and provider and patient education and training. As obstetric telehealth expands, efforts must prioritize equitable access for rural and low-income communities, so all patients can benefit from the technological advancements to support health.

## Abbreviation Used

CAH: critical access hospital
